# Editorial: Mapping the Cyberbiosecurity Enterprise

**DOI:** 10.3389/fbioe.2019.00235

**Published:** 2019-10-03

**Authors:** Randall Murch, Diane DiEuliis

**Affiliations:** ^1^Virginia Tech, Blacksburg, VA, United States; ^2^National Defense University, Washington, DC, United States

**Keywords:** cyberbiosecurity, bioeconomy, biosecurity, national security, biotechnology

We are pleased to introduce this Research Topic in Frontiers in Bioengineering and Biotechnology on a new area of biosecurity, termed “Cyberbiosecurity.” This term, originally introduced in the recently published strategic article by Murch et al. entitled “Cyberbiosecurity: An Emerging New Discipline to Help Safeguard the Bioeconomy (*Front. Bioeng. Biotechnol*. doi: 10.3389/fbioe.2018.00039), describes the security vulnerabilities that exist at the intersection of cybersecurity, cyber-physical security, and biosecurity.

Entitled “*Mapping the Cyberbiosecurity Enterprise*,” this collective of papers was amassed to firmly establish this topic as a new discipline within biosecurity. Each article contributes to developing and presenting deeper understanding of this emerging topic, and helps to delineate the range of current and potential applications of cyberbiosecurity. We also anticipate that this collective will foster greater engagement between the biosecurity and cybersecurity communities.

“Cyberbiosecurity” has been defined as “understanding the vulnerabilities to unwanted surveillance, intrusions, and malicious and harmful activities which can occur within or at the interfaces of comingled life and medical sciences, cyber, cyber-physical, supply chain and infrastructure systems, and developing and instituting measures to prevent, protect against, mitigate, investigate and attribute such threats as it pertains to security, competitiveness, and resilience.” While cybersecurity is a broad and well-researched existing field, its application to specific aspects of the life sciences necessitates a conjoining of experts from each discipline which have predominantly existed in silos to date. Defining cyberbiosecurity as a discipline is a necessary first step in bringing these disparate groups together to expand understanding of the risks from their relative perspectives.

Mapping the topology of cyberbiosecurity has just begun, but proponents have realized that it has expansive applications across the life sciences, most obviously in the biomedical and pharmaceutical domains. But as the digitization of biology grows, biotechnology is expanding far beyond these traditional silos. The purposeful engineering of biology, including application of the classical “design, build, test” cycle, is opening unprecedented opportunities for biomaterials and biofuels and their use, for agriculture and food systems (from large scale crop engineering to “farm to table”), and for bioinformatics and “AI” (from small field tools to large-scale complex systems and cloud computing). As biotechnologies continue to advance and evolve, cyberbiosecurity will be a key consideration in existing critical infrastructure related to all these arenas. Further, new components of critical infrastructure may emerge and be defined through advances in the synthetic biology industry, and cybersecurity will need to be assessed for those new components. In our view, awareness and identification of vulnerabilities is an important first step in launching the field, followed by the development and implementation of mitigations and solutions. Eventually, practitioners in this growing field will be responsible for the development of guidelines and standards of governance, which will require adherence and compatibility with existing national defense strategies.

This Special Collection, represented by both U.S. and international contributors, includes writings on a number of the topical areas described above. Vulnerabilities associated with synthetic biological manufacturing are described, including specific discussions of biopharmaceutical production. The evolving platforms for biotechnology, including distributed manufacturing models and laboratory automation, are included for consideration. Importantly, a discussion of the public health and stability ramifications of cyberbiosecurity in settings outside the US are also considered. General themes in other fields, such as agriculture, biopharma, and labs of the future are represented in stand-alone contributions. Some technical aspects of tool development, such as DNA synthesis security screens, and access to pathogen genome databases provide insights on current thinking and perceptions of risk. Finally, broad consideration is given to cyberbiosecurity in the national security context, given any new aspect of biosecurity must mesh with existing national security approaches and frameworks in the biodefense realm. Authors have also provided discussions of options for training and strategies for workforce development, all of which can help to build not only a general awareness of cybersecurity among biologists and synthetic biology engineers, but potentially develop a core of cyberbiosecurity specialists or practitioners that will be needed for risk assessments and solutions.

It is our hope that this eclectic set of insights and perspectives will broadly stimulate academia, government, non-profits, and the private sector to identify, prioritize, resource and pursue research, and implement solutions in the realm of cyberbiosecurity. Such research, outcomes and change management should focus on risk analysis, methods and technologies, education and training, guidelines and standards, policy, regulations and legal frameworks.

## Author Contributions

All authors listed have made a substantial, direct and intellectual contribution to the work, and approved it for publication.

### Conflict of Interest

The authors declare that the research was conducted in the absence of any commercial or financial relationships that could be construed as a potential conflict of interest.

